# Characterization of Transcriptional Complexity during Adipose Tissue Development in Bovines of Different Ages and Sexes

**DOI:** 10.1371/journal.pone.0101261

**Published:** 2014-07-01

**Authors:** Yang Zhou, Jiajie Sun, Congjun Li, Yanhong Wang, Lan Li, Hanfang Cai, Xianyong Lan, Chuzhao Lei, Xin Zhao, Hong Chen

**Affiliations:** 1 College of Animal Science and Technology, Northwest A&F University, Shaanxi Key Laboratory of Agricultural Molecular Biology, Yangling, Shaanxi, China; 2 United States Department of Agriculture–Agricultural Research Service, Bovine Functional Genomics Laboratory, Beltsville, Maryland, United States of America; 3 Institute of Cellular and Molecular Biology, Jiangsu Normal University, Xuzhou, Jiangsu, China; Wageningen UR Livestock Research, Netherlands

## Abstract

**Background:**

Adipose tissue has long been recognized to play an extremely important role in development. In bovines, it not only serves a fundamental function but also plays a key role in the quality of beef and, consequently, has drawn much public attention. Age and sex are two key factors that affect the development of adipose tissue, and there has not yet been a global study detailing the effects of these two factors on expressional differences of adipose tissues.

**Results:**

In this study, total RNA from the back fat of fetal bovines, adult bulls, adult heifers and adult steers were used to construct libraries for Illumina next-generation sequencing. We detected the expression levels of 12,233 genes, with over 3,000 differently expressed genes when comparing fetal and adult patterns and an average of 1000 differently expressed genes when comparing adult patterns. Multiple Gene Ontology terms and pathways were found to be significantly enriched for these differentially expressed genes. Of the 12,233 detected genes, a total of 4,753 genes (38.85%) underwent alternative splicing events, and over 50% were specifically expressed in each library. Over 4,000 novel transcript units were discovered for one library, whereas only approximately 30% were considered to have coding ability, which supplied a large amount of information for the lncRNA study. Additionally, we detected 56,564 (fetal bovine), 65,154 (adult bull), 78,061 (adult heifer) and 86,965 (adult steer) putative single nucleotide polymorphisms located in coding regions of the four pooled libraries.

**Conclusion:**

Here, we present, for the first time, a complete dataset involving the spatial and temporal transcriptome of bovine adipose tissue using RNA-seq. These data will facilitate the understanding of the effects of age and sex on the development of adipose tissue and supply essential information towards further studies on the genomes of beef cattle and other related mammals.

## Introduction

The role of adipose tissue in development is extremely complex and important. In addition to providing insulation and mechanical support, adipose tissues have been traditionally defined as the major sites for the storage of surplus fuel, and they provide fuel to generate energy [1]. Recently, adipose tissue has been emerging as an active participant in the regulation of physiologic and pathologic processes [2]. Adipose tissue has been identified as a key component of the endocrine system and the immune system because many adipose-derived secreted products, including hormones and inflammatory cytokines, are detected in this tissue [3], [4]. In humans, the excessive growth of adipose tissue, also know as obesity, has long been known as an independent risk factor for human health conditions, such as myocardial infarction, stroke, type 2 diabetes mellitus, and certain cancers [5]–[7]. As the prevalence of obesity appears to be increasing on a global level, the role of adipose tissue in metabolic health conditions has gained significant attention. However, in cattle, adipose tissue is the most important tissue in lipid synthesis and not only serves in the aforementioned functions but also plays a key role in the quality of beef [8]. In Asia, snowflake beef is very popular for its beautiful texture and excellent taste, while it is considered to be healthier to eat beef with little fat in many other countries. Nevertheless, the amount of fat in beef is already of interest. To date, many in vitro studies have analyzed adipogenesis, and research progress has increased the knowledge of complex molecular mechanisms during development, but a systematic study of adipose tissue at the transcriptional level is necessary.

Age and sex are two key factors that can affect the development of white adipose tissue. During late gestation and the first postnatal weeks, white adipose tissue emerges and begins to further develop [9]. In the juvenile period, the degree of body fat mass is characterized by the number or size of fat cells or a combination of both [10]. After sexual maturation, the number of white adipose tissue cells is nearly stable, and the cause of increasing adipose tissue mass is mainly due to increased triglyceride accumulation [11]. Both testosterone and estrogen regulate the amount of white adipose tissue [12], [13]. Testosterone and dihydrotestosterone inhibit the adipogenic differentiation of preadipocytes by molecular mechanisms involving activation of the AR/β-catenin interaction [14]. In Heine's study, the absence of ERα caused adipocyte hyperplasia and hypertrophy in both sexes [12]. Furthermore, studies have shown that castration improves the fat deposition capability of males and that ovariectomy increases adipose deposition, which is then reversed by estrogen replacement [14]–[16]. In cattle, steers have a faster rate of fat deposition than bulls and show better marbling scores [17]. Therefore, by comparing the adipose tissues of individuals at various ages and sexes, we hope to identify differences in transcriptional expression profiles. These data would be a significant contribution towards the understanding of the development of adipose tissue and lipid accumulation and will therefore contribute to the study of obesity in humans and help to control the amount of fat in beef.

High-throughput sequencing of RNA (RNA-Seq) is an efficient way of mapping and quantifying transcriptomes and was developed to help analyze global gene expression in different tissues. In comparison to a microarray approach, which can be limited by the number of probes on the array, RNA-Seq analyzes all genes expressed in a given tissue [18]. Here, we constructed the transcriptional profile of bovine adipose tissue at a global level, compared the genes expressed in the subcutaneous adipose tissues of Chinese Qinchuan fetal bovines, adult bulls, adult heifers and adult steers, and analyzed the effects of age and sex on the expressional level of genes in adipose tissue. This study will provide essential information in support of further research on adipose tissue.

## Results

### RNA sequencing of bovine subcutaneous adipose tissues

**Figure 1:**
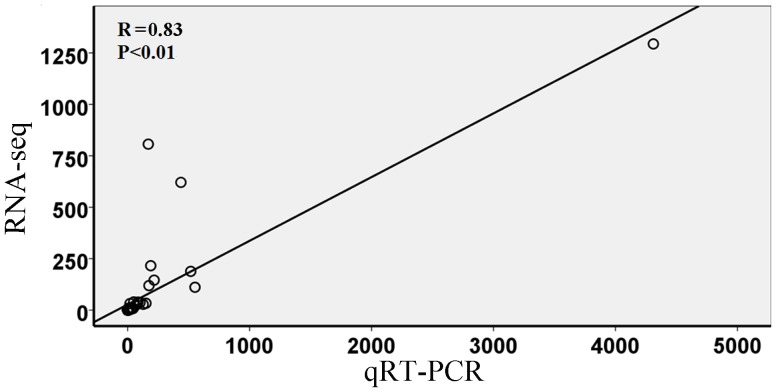
Verification of gene expression analysis by quantitative realtime PCR (qRT-PCR). Note: Individual gene expression ratios were calculated using RPKM data generated by RNA-seq and plotted against calculations done for the same gene using qRT-PCR.

To obtain a global view of the bovine adipose tissue transcriptome, total RNA from the subcutaneous adipose tissues of fetal bovines, adult bulls, adult heifers and adult steers (ATFB, ATAB, ATAH and ATAS) were used to construct RNA libraries for Illumina sequencing. In total, we acquired an average of 27 billion clean reads from each library. Approximately 80% of the total reads uniquely mapped to the reference genome (http://hgdownload.soe.ucsc.edu/goldenPath/bosTau7/bigZips/bosTau7.fa.masked.gz). A total of 3% of sequences matched multiple positions in the reference genome, and 17% did not map to the reference genome (Table 1). Only the uniquely mapped reads were considered in this analysis. Gene expression level was calculated using the reads per kilobase transcriptome per million mapped reads (RPKM) method. To validate the RNA-Seq results, 15 genes that were uniquely or highly expressed in one of the four samples were selected and normalized to β-actin via the qRT-PCR method (Figure S1). We calculated the correlation efficiency of qRT-PCR and RNA-seq data using SPSS software (version 1.70), and the result (Figure 1) supported our sequence data (P<0.01, R = 0.83).

**Table 1:** Summary of sequence read alignments to the reference genome.

	ADFB		ADAB		ADAH		ADAS	
Map to Genome	reads number	percentage	reads number	percentage	reads number	percentage	reads number	percentage
Total Reads	26565078	100.00%	27103680	100.00%	27023928	100.00%	26333936	100.00%
Total BasePairs	2390857020	100.00%	2439331200	100.00%	2432153520	100.00%	2370054240	100.00%
Total Mapped Reads	21937600	82.58%	22399630	82.64%	22812043	84.41%	21900722	83.17%
perfect match	14816158	55.77%	14342188	52.92%	16383118	60.62%	14730449	55.94%
≦5 bp mismatch	7121442	26.81%	8057442	29.73%	6428925	23.79%	7170273	27.23%
unique match	20448151	76.97%	21579397	79.62%	22044040	81.57%	21119926	80.20%
multi-position match	1489449	5.61%	820233	3.03%	768003	2.84%	780796	2.96%
Total Unmapped Reads	4627478	17.42%	4704050	17.36%	4211885	15.59%	4433214	16.83%

Note: ATFB: adipose tissues of fetal bovines; ATAB: adipose tissues of adult bulls; ATAS: adipose tissues of adult steers; ATAH: adipose tissues of adult heifers.

### Analysis of Gene Expression in Adipose tissue

In our study, we detected a total of 12,233 genes expressed in the four samples. To better categorize these genes, which presented differential expression levels, gene expression RPKM values were categorized into three groups: high (≥500 RPKM), medium (10 to 500 RPKM), and low (<10 RPKM) (Table 2). There were 184 (1.60%), 181 (1.58%), 169 (1.45%) and 167 (1.44%) genes highly expressed in ATFB, ATAB, ATAH and ATAS, respectively. Genes that are highly expressed in a specific tissue may be responsible for the basic metabolism and function of that tissue. In the four expression patterns that were detected, 109 genes were expressed commonly, among which 63 genes were related to the protein components of ribosomes, and all of these genes were more highly expressed in ADFB than in the adipose tissue of adult cattle (Table S1). To ascertain the other potential functions of adipose tissue, we performed GO analysis for the other 46 genes. These results showed that those genes were involved in various cell component, molecular function and biological processes, and no GO term was significantly enriched (P<0.05) (Figure S2).

**Table 2:** RNA-Seq gene expression results for the four patterns of adipose tissues.

Category	ATFB	ATAB	ATHB	ATAS
Highly expressed genes♯	172	135	107	112
Medium expressed genes§	7,770	7,101	8,394	8,664
Lowly expressed genes∮	23,294	23,767	23,382	22,732
Total expressed genes	31,236	31,003	31,883	31,508
Unexpressed genes	5,137	5,370	4,490	4,865

Note: ATFB: adipose tissues of fetal bovines; ATAB: adipose tissues of adult bulls; ATAS: adipose tissues of adult steers; ATAH: adipose tissues of adult heifers.

♯ Gene expressional level ≥500 RPKM

§ Gene expressional level ranging from 10 to 500 RPKM

∮ Gene expressional level <10 RPKM.

**Figure 2:**
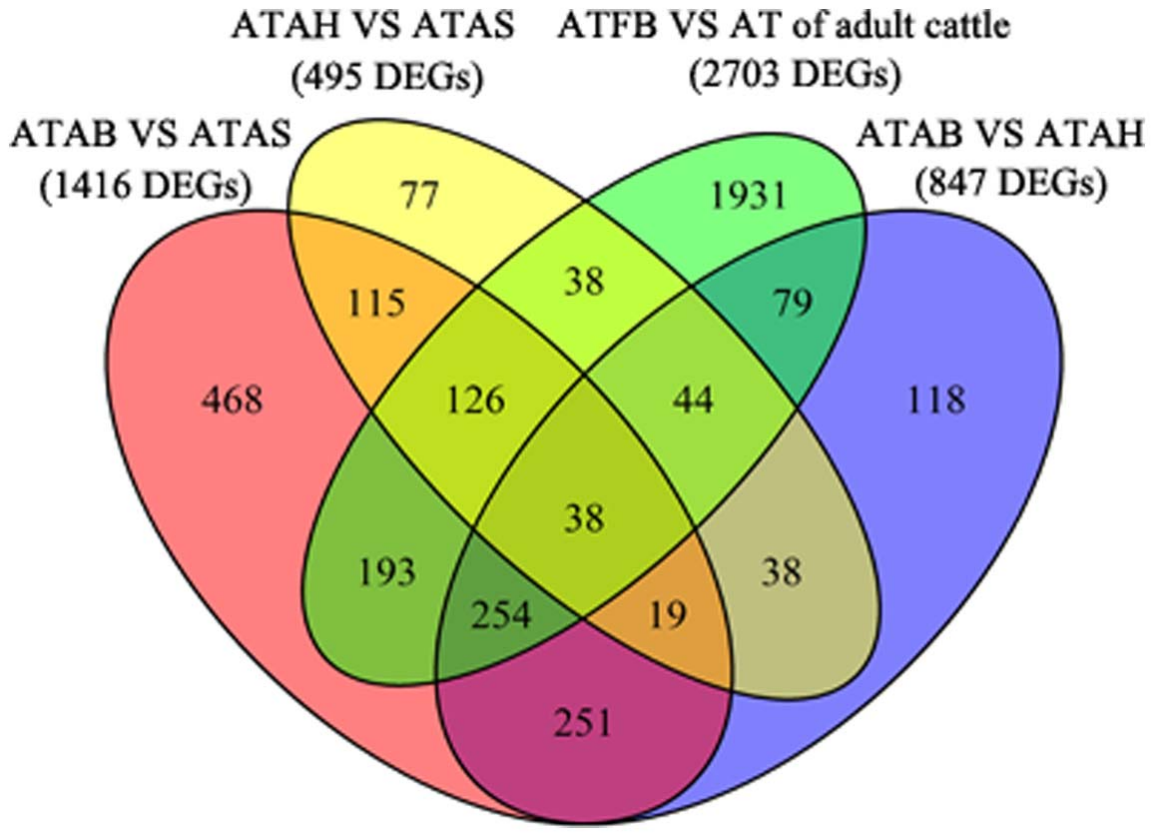
Distribution of differential expression genes among the four adipose tissues. Note: ATFB: adipose tissues of fetal bovines; ATAB: adipose tissues of adult bulls; ATAS: adipose tissues of adult steers; ATAH: adipose tissues of adult heifers; AT: adipose tissues

Age plays an important role in the development of adipose tissue. We compared the expressional differences in adipose tissue between fetal bovine at 200∼250 days, at which time the white adipose tissue just emerges and its development starts, and adult cattle at 20 months, whose adipose tissue increases mainly due to the accumulation of triglycerides. In this study, the expression profiles of adipose tissue changed considerably from fetuses to adults. There were 3,188, 4,167 and 3,903 genes differently expressed in ATAB, ATAS and ATAH compared to ATFB, respectively. We selected a total of 2,703 genes that were differentially expressed between the two stages using the edgeR method [19]. During development, sex is not only an important turning point in metabolism, but is also a key factor resulting in individual differences. To detect the effect of sex on adipose tissue development, we compared the expression profiles of adipose tissues in adult bulls, adult cows and adult steers at 20 months of age. A total of 847 genes were found to differ significantly in expressional levels between ATAB and ATAH. The number of DEGs between ATAS and ATAB (1,464 DEGs) was approximately three times higher than between ATAS and ATAH (495 DEGs). When mixed together, there were 57 genes differentially expressed among the three adipose tissues of adult cattle and 38 genes differentially expressed among all the four adipose tissues. The distribution of differential expression genes among the four adipose tissues was shown in Figure 2.

### Gene Ontology Analysis of the DEGs

To further investigate the biological processes associated with the 2,703 differentially expressed genes, we performed Gene Ontology (GO) analysis by running queries for each differentially expressed gene against the GO database, which provides information related to three ontologies: molecular function, cellular component, and biological process. First, we arranged GO analysis for DEGs between ATFB and adult bovine adipose tissue, and the results are shown in Table S2. In total, we found that 2979 GO terms were assigned to the DEGs, and among these genes, there were 251 GO terms corresponding to molecular function, 519 GO terms corresponding to cellular component and 2209 GO terms corresponding to biological process. Structural molecule activity and receptor activity were the only two significantly enriched molecular functions. In the cellular component category, 9 terms were significantly enriched (P<0.05). The four most significantly enriched GO terms included the structural constituents of ribosomes, followed by genes expressed in the extracellular region, ribonucleoprotein complex, extracellular matrix, and genes that are integral to membrane structures. In the case of biological processes, 22 GO terms were significantly enriched and were related to various processes, such as organ development, cell proliferation, and the immune system. Lipid metabolic processes were not found to be enriched significantly, with a comparatively low corrected P-value (0.053).

GO analysis was performed for DEGs between the adipose tissues of male and female cattle (Table S3–S5). When comparing ATAB and ATAS, we detected 2311 GO terms, including 193 GO terms in the molecular function category, 400 GO terms in the cellular component category and 1718 GO terms in the biological process category. In the molecular function category, GO terms for carbohydrate derivative binding and receptor activity were significantly enriched. There were 9 GO terms in the molecular function category that were significantly enriched, which were mostly related to the cell membrane, especially to the mitochondrial membrane. In the biological process category, we found 33 GO terms that were significantly enriched, including the GO terms that we expected, such as steroid hormone stimulus and response to lipids. Three GO terms related to cytokines were also significantly enriched.

When comparing ATAB and ATAH, we detected 1762 GO terms, including 148 GO terms in the molecular function category, 283 GO terms in the cellular component category and 1330 GO terms in the biological process category. In the molecular function category, there were 7 significantly enriched GO terms. Apart from the GO terms that were significantly enriched between ATAB and ATAS, cytokine activity, signal transducer activity, molecular transducer activity, motor activity and transmembrane signaling receptor activity were also significantly enriched. In the cellular component category, the DEGs that were most enriched included those categorized to the extracellular region. In the biological process category, we found 18 GO terms that were significantly enriched. Similar to the comparison between ATAB and ATAS, GO terms related to steroid hormone stimulus, response to lipids and cytokines were significantly enriched.

Comparing ATAH and ATAS, we detected 1310 GO terms, including 125 GO terms in the molecular function category, 215 GO terms in the cellular component category and 970 GO terms in the biological process category. In the molecular function category, the most significantly enriched were carbohydrate derivative binding, followed by oxidoreductase activity, transferase activity and cell surface binding. The DEGs were significantly enriched in cellular components of the extracellular matrix, intrinsic to membrane, extracellular region part and in the extracellular region. In the biological process category, 21 GO terms were significantly enriched. Compared to the other two comparisons between adipose tissues of adult bovine, GO terms related to steroid hormones were also included in the 21 GO terms, and more processes related to lipid metabolism were significantly enriched, including the following: fatty-acyl-CoA metabolic process, lipid metabolic process, neutral lipid metabolic process, acylglycerol metabolic process, acyl-CoA metabolic process, triglyceride metabolic process and regulation of the lipid biosynthetic process. The estrogen stimulus response process was also found to be significantly enriched, while no GO term related to cytokines was found to be significantly enriched.

### KEGG Pathway Analysis of the DEGs

Different genes usually cooperate with each other to exercise their biological functions. Pathway-based analysis helps to further understand the biological functions of genes. Overall, 228 pathways (17 pathways significantly enriched, P<0.05) were assigned to the DEGs in the comparison of ATFB and adipose tissue from adult bovines (Table S6). The most significantly enriched pathway was ribosomal. The PPAR signaling pathway, one the most important pathways related to the development of adipose tissue, was also found to be significantly enriched. Comparing ATAB and ATAS, 201 pathways (16 pathways significantly enriched, P<0.05) were assigned to the DEGs (Table S7). The pathway term showing the highest level of significance was type I diabetes mellitus, followed by oxidative phosphorylation, pathways in cancer, ECM-receptor interaction, proximal tubule bicarbonate reclamation, p53 signaling pathway, sphingolipid metabolism and cytokine-cytokine receptor interaction. Comparing ATAB and ATAH, we detected 183 pathways (13 pathways significantly enriched, P<0.05) assigned to the DEGs (Table S8). Type I diabetes mellitus and cytokine-cytokine receptor interaction were also among the significantly enriched pathways. In the comparison between ATAH and ATAS, there were 170 pathways (12 pathways significantly enriched, P<0.05) assigned to the DEGs (Table S9). Steroid biosynthesis, instead of type I diabetes mellitus, was the most significantly enriched pathway. Furthermore, biosynthesis of unsaturated fatty acids, fatty acid elongation and the PPAR signaling pathway also showed significance in our analysis.

### Differential alternative splicing in adipose tissue

The study of alternative splicing (AS) has long been a valuable subfield of molecular biology, but this subfield was not given enough attention until recent years. AS plays a major role in the generation of proteomic and functional complexity in higher organisms [20]. In our data, we detected 12,233 genes, of which up to 4,753 genes underwent AS events. Therefore, in total, approximately 38.85% of the genes in adipose tissues were found to be alternatively spliced in our study. However, single-sample analysis showed that more than 50% of the AS events were specifically expressed in the adipose tissues we studied, thereby indicating a strong association of these AS events with the regulation of adipose tissues in specific physiological environments. Taking this phenomenon into consideration, it must be noted that the number of genes that underwent AS events in adipose tissues may have been far higher than 4,753 because a single tissue can encounter various physiological environments during development. These data suggested a major role for AS in contributing to the complexity of gene expression in adipose tissue.

**Figure 3:**
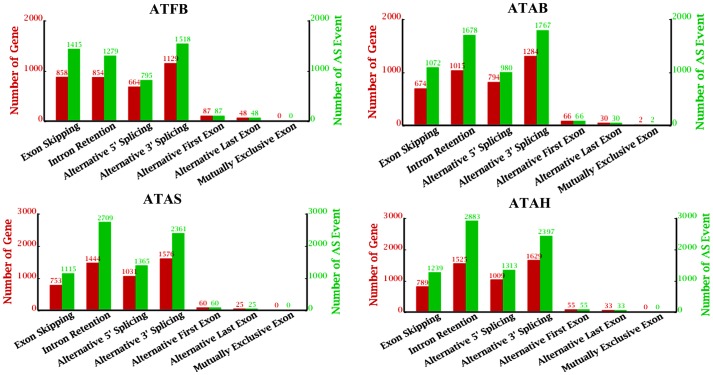
Statistic charts of alternative splicing events and genes in the four adipose tissues. Note: ATFB: adipose tissues of fetal bovines; ATAB: adipose tissues of adult bulls; ATAS: adipose tissues of adult steers; ATAH: adipose tissues of adult heifers.

To identify the types of AS mechanisms being used by genes expressed in adipose tissues, we conducted an analysis of AS events in the four types of adipose tissue (Figure 3). All seven known types of AS models were found in ATAB. These seven types included exon skipping (ES), intron retention (IR), alternative 5′ splice site (A5SS), alternative 3′ splice site (A3SS), alternative first exons (AFE), alternative last exons (ALE) and mutually exclusive exons (MXE). In the other three adipose tissues, there were six known types of AS models. Here, no AS events were categorized as the mutually exclusive exon model, and this type of AS was only found twice in ATAB. Almost all AS events in the four adipose tissues belonged to the four models: ES, IR, A5SS, A3SS, and only a few AS events occurred in the other three models. However, analysis of the distributions of the main four AS models highlighted a few differences between ADFB and the adipose tissue of adult cattle: ES and A3SS had a comparative advantage in ADFB, while IR and A3SS were more abundant than ER and A5SS in ADAC.

### Identification of novel transcribed units

The current gene annotation is almost exclusively based on gene prediction programs that detect protein coding regions and do not include gene coding for small proteins (<100 codons) [21]. Non-coding RNAs (ncRNAs) appear to comprise a hidden layer of internal signals that control various levels of gene expression in physiology and development [22]. However, very few ncRNAs, with the exception of microRNAs, have been found and analyzed in adipose tissue to date. To identify novel transcription units, we compared the distribution of assembled reads with the annotation of reference genes using stringent criteria. In total, 4,020, 4,539, 4,713, and 4,183 novel transcription units (NTUs) were found in ATAB, ATAS, ATAH, and ATAF, respectively. The Coding Potential Calculator was used to predict the coding ability of the NTUs. The majority of the NTUs were not expected to encode proteins, and only approximately 30% of the NTUs were considered to have coding ability. Of the NTUs with coding ability, 45, 43, 46, and 31 NTUs had fewer than 100 codons in ATAB, ATAS, ATAH, and ATAF, respectively. Next, we analyzed the distribution of all of the NTUs. First, the increase in the number of NTUs per 1,500 bp was calculated and suggested two basic clusters of tendency according to the coding ability of the NTUs. In table 3, the numbers of NTUs under 1,500 bp both with and without coding ability occupied over 50% in the four samples and decreased as an index per 1,500 bp. This illustrated that both NTUs with and without coding ability were enriched under1,500 bp and that the main difference in the numbers of the two types of NTUs was also concentrated in this range.

**Table 3:** Distributions of the novel transcript units with and without coding ability.

Length (bp)	Number of NTUs with coding ability				Number of NTUs without coding ability			
	ATFB	ATAB	ATAH	ATAS	ATFB	ATAB	ATAH	ATAS
<1,500	578	684	730	674	1937	1572	1711	1626
1,500–3,000	263	319	362	354	793	640	723	688
3,000–4,500	137	175	243	243	287	246	372	360
4,500–6,000	43	104	126	129	81	133	194	204
6,000–7,500	9	38	62	72	29	55	76	85
7,500–9,000	5	19	30	21	8	10	31	29
9,000–10,500	2	7	9	11	7	12	20	21
10,500–12,000	0	2	5	7	0	2	6	3
12,000–13,500	2	1	2	3	0	0	4	3
13,500–15,000	0	0	0	2	0	0	0	2
15,000–16,500	0	0	0	0	0	1	1	0
16,500–18,000	0	0	0	0	1	0	0	1
18,000–19,500	0	0	0	0	0	0	2	0

Note: ATFB: adipose tissues of fetal bovines; ATAB: adipose tissues of adult bulls; ATAS: adipose tissues of adult steers; ATAH: adipose tissues of adult heifer.

### Detecting SNP Variants in Pooled Transcriptome Samples by RNA-Seq

RNA-seq has been shown to be an efficient way to detect SNP variants on a large scale at the mRNA level. In bovine milk, Angela Cánovas and his colleagues discovered 100,734 SNPs in Holstein samples using RNA-seq and validated these results by Sanger sequencing technologies, which confirmed that RNA-Seq technology is an efficient and cost-effective method to identify SNPs in transcribed regions [23]. In our study, to detect the SNPs at the transcriptome level, we applied SOAPsnp software and detected 56,564, 65,154, 78,061 and 86,965 putative SNPs in ATFB, ATAB, ATAH and ATAS, respectively. These results are shown in Table_S10 to Table_S13.

## Discussion

To date, the RNA-Seq method has been widely used to detect DEGs between two compared patterns or gene expression patterns, NTUs, AS events and SNPs for a single tissue or cell lines. In bovines, the profiles of milk and mammary tissue at different stages of lactation and the profiles of muscle at different stages were recently collected and compared using the RNA-Seq method [24]–[26]. However, our current knowledge on the development of bovine adipose tissue has been limited. Age and sex are two key factors that affect the development of white adipose tissue. Herein, we present, for the first time, a complete dataset detailing the spatial and temporal transcriptome of bovine adipose tissue using RNA-seq.

**Figure 4:**
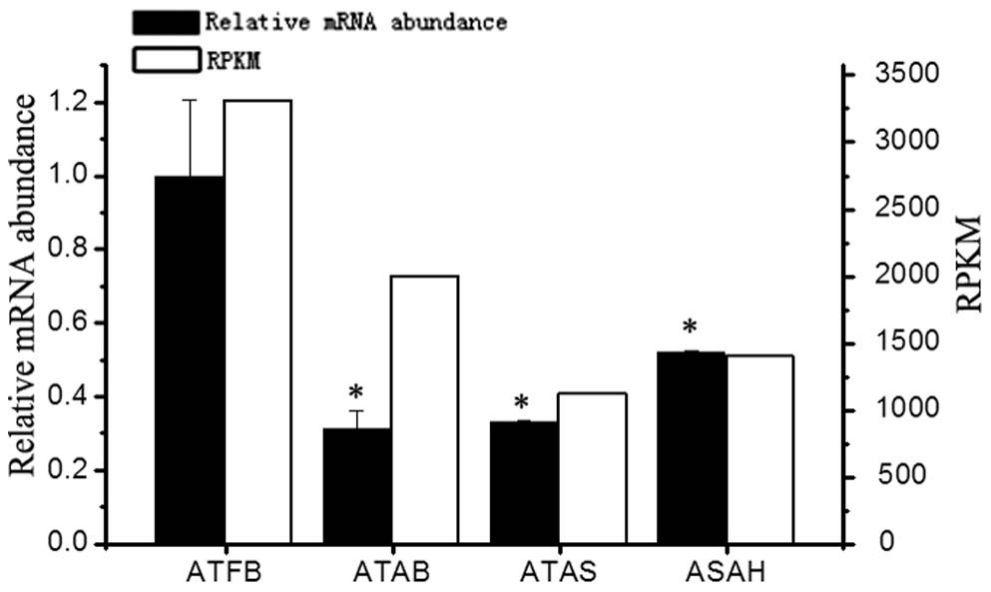
The expression of GAPDH in the four adipose tissues detected by RT-qPCR and RNA-seq. Note: ATFB: adipose tissues of fetal bovines; ATAB: adipose tissues of adult bulls; ATAS: adipose tissues of adult steers; ATAH: adipose tissues of adult heifers. *: P<0.05

White adipose tissue has long been widely recognized as the major site for the storage of surplus fuel or fuel to generate energy. In our study, we found 109 genes expressed over 500 RPKM, among which 63 genes were relative to protein components of ribosomes. Ribosome proteins are responsible for the basic biochemistry of protein synthesis. To date, over 30 adipokines have been found in white adipose tissue, most of which are proteins [2], [27], [28]. This finding illustrates that adipose tissue may not only store surplus fuel but may also be an extremely active tissue for protein metabolism. Several genes related to lipid metabolism were found, including GAPDH, APOE and FABP4. Notably, GAPDH, a common housekeeping gene, was expressed at significantly different levels between ATFB (3308.42 RPKM) and both ATAC (1403.75 RPKM) and ATAS (1127.50 RPKM). To confirm this, we validated the GAPDH expression levels in the four samples using qPCR method. The result showed that the GAPDH expression levels in all the adult samples were significantly lower (P<0.05) than the fetal sample and no significant difference was seen among adult samples (Figure 4). Those suggested that GAPDH may not be used as a housekeeping gene in adipose tissue over different stages while also supporting the critical role of GAPDH in adipose tissue.

**Figure 5:**
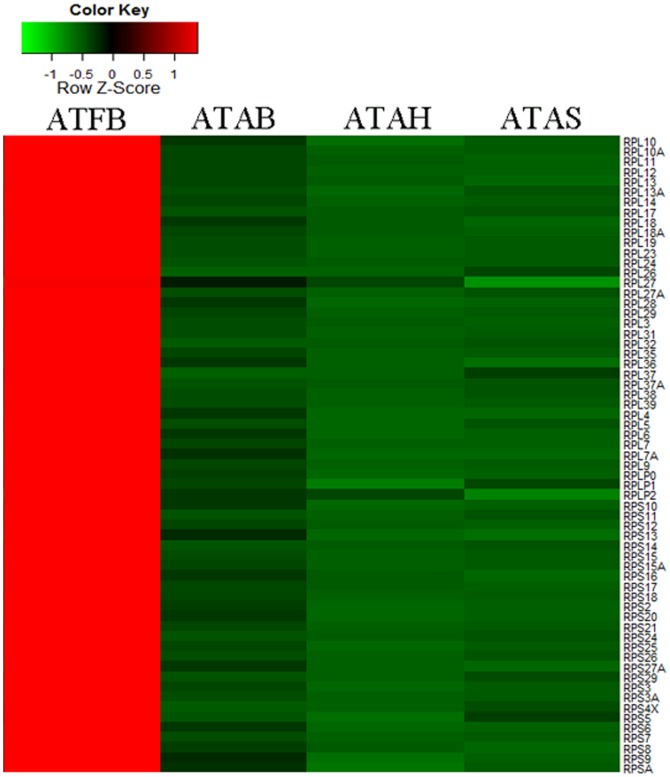
Changes of the expression levels of genes relative to the protein components of ribosome. Note: ATFB: adipose tissues of fetal bovines; ATAB: adipose tissues of adult bulls; ATAS: adipose tissues of adult steers; ATAH: adipose tissues of adult heifers.

**Figure 6:**
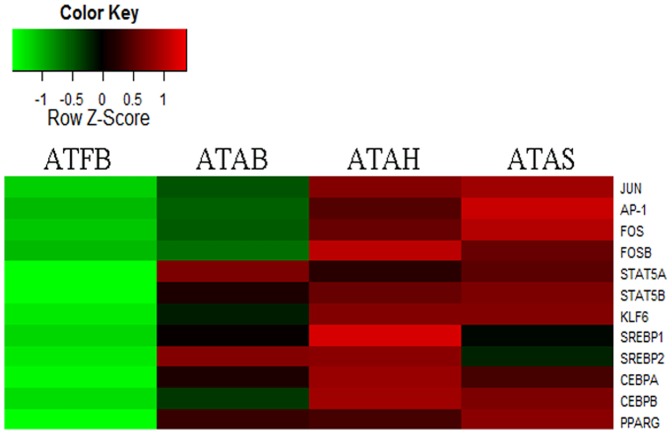
Changes of the expression levels of genes playing a positive role in the development of white adipose tissue. Note: ATFB: adipose tissues of fetal bovines; ATAB: adipose tissues of adult bulls; ATAS: adipose tissues of adult steers; ATAH: adipose tissues of adult heifers.

The number of DEGs changed considerably from fetuses to adults in our study. We performed GO and pathway analysis for the DEGs. Organ development and cell proliferation were the two most significantly enriched biological processes. He et al compared the muscle profiles between newborn and 30 month cattle, and the most important enriched biological processes were found to be related to developmental process and multicellular organism development [26]. The development and function of one tissue type is perfected with increasing age. Both of the most significantly enriched cellular components and pathways were related to ribosomes. Additionally, the 63 highly expressed genes related to the protein components of ribosomes showed an absolute accordance in that their expression levels were all reduced in the adipose tissue of fetuses compared to adults (Figure 5). These data suggest that protein synthesis in bovine adipose tissue may be more active in fetuses than in adults. The well known transcription factor PPARG, along with members of the C/EBP, STAT, SREBP, KLF AP-1 families, has been shown to play a positive role in the development of white adipose tissue [29]. We analyzed the 18 genes involved in those important families, among which, 12 genes demonstrated an upward trend from fetuses to adults (Figure 6). Apart from those genes, pathway analysis found that the insulin and PPAR signaling pathways were significantly associated with DEGs in both ATFB and the adipose tissues of adult cattle. The PPAR signaling pathway is regarded as an extremely important pathway in adipose tissue [30]. There were 22 genes in the PPAR signaling pathway that were significantly changed (Figure S3). The expression levels of the two major transcriptors (PPARG and PPARD) increased significantly in adipose tissue from the fetal period to adulthood. Genes related to cell survival, ubiquitination and adaptive thermogenesis processes were not found to differ significantly in expression level and were thus concluded to be important in the fundamental survival of adipose tissue. The expression of genes related to lipid metabolism, adipocyte differentiation and gluconeogenesis were mostly up-regulated. In the lipid transport process, Apo-AI and Apo-AII, which are known to contribute to lipid storage, were down-regulated in adipose tissue from adult bovines. Taken together, these data suggest that lipid metabolism is more active in the adipose tissue of adult bovines than in fetal bovines.

Studies of the differences between the adipose tissues of male and female adult cattle have mainly focused on comparing ATAB and ATAS because of the wide use of castration in cattle cultivation. However, it has been proven that both androgens and estrogens regulate the amount of white adipose tissue. Therefore, it is necessary to compare the genes expressed in ATAB, ATAS and ATAH. The number of DEGs between ATAH and ATAS was the least among the three comparisons, as this value was only one third of the number of DEGs between ATAB and ATAS. This result suggests that the gene expression profile in the adipose tissues of bulls following castration may change and become more like that of the expression profile in the adipose tissue of heifers. GO and pathway analysis showed that 7 GO terms and 2 pathways related to lipid metabolism were significantly enriched, thereby demonstrating that lipid metabolism in ATAH and ATAS were dramatically different. Previous studies have proven that castration improves males' capability for fat deposition [31]. In our study, the steroid hormone stimulus response process and lipid response process were significantly enriched in the GO analysis for the DEGs between ATAB and ATAS. Castration reduced the production of testosterone, which combines with androgen receptor (AR) to fulfill its functions [32]. AR has been found in the adipose tissues of a wide range of species, including humans, mice, and cattle, where it has been shown that testosterone may directly effect the development of adipose tissue [33], [34]. Experiments with cultured adipocytes have also demonstrated the important functions of androgen in adipogenesis [35], [36]. However, found no significant difference in the expressional level of AR between ATAB and ATAS. Choi et al found that steers showed better marbling scores and better quality grades but also detected no difference between the AR mRNA levels in the adipose tissues of Korean bulls and steers [33]. Besides, estrogen receptor (ESR) was also expressed in the four adipose tissues whereas its expression level, just like ER, did not saw significant difference between ATAB and ATAS. Thus, the greater capacity for fat deposition in ATAS may be caused by a decrease of sex hormone rather than the decrease of their receptor levels.

**Figure 7:**
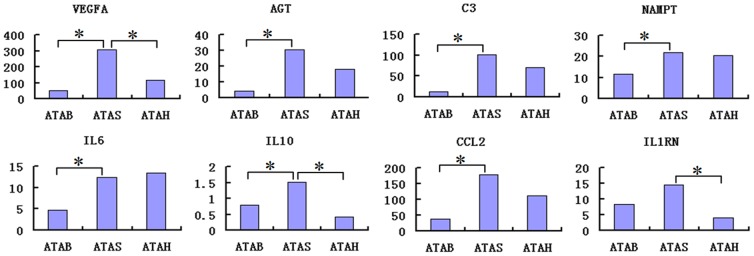
Changes of the expression levels of genes relative to adipocyte-derived proteins in adipose tissue of adult cattle. Note: Y-axis: expression level (RPKM); ATFB: adipose tissues of fetal bovines; ATAB: adipose tissues of adult bulls; ATAS: adipose tissues of adult steers; ATAH: adipose tissues of adult heifers.

As the most important tissue for lipid synthesis in cattle, adipose tissues are also regarded as an active endocrine organ. The integrated relationship between endocrine function and obesity is currently a subject of much research interest [37]. The leptin protein, one of the most abundant adipokines in adipose tissues, is central to the regulation of energy metabolism in mammals [38]. Furthermore, inflammatory markers, such as C-reactive protein (CRP) and IL-6, are found at higher levels in obese individuals than in lean subjects [39], [40]. In our study, GO terms and pathways related to cytokines were significantly enriched for the DEGs between the three adipose tissues of adult cattle. We analyzed the mRNA expression levels of 32 adipocyte-derived proteins and found that 8 genes (VEGFA, AGT, C3, NAMPT, IL6, IL10, CCL2 and IL1RN) exhibited significantly different expression among the three adult adipose tissue patterns (Figure 7). All 8 adipokines exhibited significantly higher expression in ATAS than ATAB. Therefore, we suggest that adipokines may play some role in the differential regulation of adipose tissue development in male and female cattle sexes.

In adipose tissue, only several alternatively spliced genes have been studied so far. The study of alternative splicing has long been a valuable subfield of molecular biology, and it is considered to be a key factor underlying increased cellular and functional complexity in higher eukaryotes [41]. Based on deep sequencing methodologies, current estimates indicate that more than 90% of human genes have at least two transcript isoforms [42]. This result presents a very good reference for AS events that occur in adipose tissue. However, it is worth noting that a considerable number of AS events exist uniquely in different tissues, resulting in various gene functions in different tissues. As the master regulator of adipogenesis, PPARG is expressed as two isoforms, PPARG1 and PPARG2. PPARG1 is expressed in many tissues, whereas PPARG2 expression is restricted almost exclusively to adipose tissue [43]. Pan's mRNA-Seq datasets for the whole brain, cerebral cortex, heart, skeletal muscle, lung and liver showed that each tissue dataset contributed between 18% and 31% of the detected known junctions and that many of the splice junctions were tissue-specific [44]. This version is not limited to mammals. In rice, the expression analysis of alternative splicing showed that 59% of the AS events were organ-specific, indicating a strong association of AS events with organ-specific regulation [44]. In our study, a strong association of AS events with adipose tissues of specific ages and sexes were also detected, with more than 50% of the AS events specifically expressed in each pattern. This means not only that a variety of AS events occurred in different tissues but that there is complexity in the regulation of their development through the expression of different isoforms in different physiological environments. Therefore, it is essential and urgent to distinguish the function of one gene from those of its various transcript isoforms to better understand the molecular mechanisms of adipose tissue development.

We also supplied a large amount of data regarding both NTUs and SNPs in adipose tissue, which will contribute to further understanding of its development and functions. Long non-coding RNAs (lncRNAs) are RNAs without coding ability that exceed 200 bp in length [45]. Recently, lncRNAs have emerged as a new paradigm in epigenetic remodeling [46]. It has been demonstrated that lncRNAs are essential regulators of a variety of biological processes [47]–[50]. To date, the global expression patterns of lncRNAs in adipose tissues have not been explored. In this study, numerous NTUs were detected, and two-thirds of these NTUs were considered to lack coding ability. Our study provides an abundant resource for the further study of lncRNAs in adipose tissue.

In conclusion, we analyzed the expression profiles of bovine subcutaneous adipose tissue samples taken at different ages and sexes for the first time. A huge number of DEGs for each compared-pair, AS events, NTUs and SNPs were detected in adipose tissue. We described the effects of age and sex on the development of adipose tissue using GO and pathway analysis. By comparing adipose tissues at different stages, we found that protein synthesis was more active in ATFB, whereas lipid metabolism was more active in the adipose tissues of adult bovines. As for the affect of sex, we conjectured that adipokines may be one of the factors responsible for the different development of adipose tissue in different sexes. We also analyzed the distributions of AS events, NTUs and SNPs, along with the analysis of DEGs, which not only revealed new and old information regarding bovine adipose tissue development but also supplied a new understanding of adipose tissue at a molecule level.

## Methods

### Ethics statement

All animals in this study were approved by Institutional Animal Care and Use Committee (IACUC) of Northwest A&F University and Qinbao Animal Husbandry Co., Ltd, respectively. All surgery was performed under sodium pentobarbital anesthesia, and all efforts were made to minimize suffering. Bovine embryos of slaughtered cows were collected from the Tumen abattoir, a local slaughterhouse of Xi'an, P.R. China. Heifers, bulls, and steers were obtained from Shanxi Kingbull Livestock Co., Ltd.

### Sample collection and RNA sequencing

Bovine embryos at approximately 250 days (gestation period 280 days) were collected from the reproductive tracts of slaughtered cows. The adult animals were kept in free-stall housing, fed total mixed ration, and slaughtered at 20 months. In this study, five bovine embryos, five adult cows, five adult bulls, and five adult steers of Qinchuan cattle were selected. The subcutaneous adipose tissues were collected immediately after the cattle were slaughtered, snap-frozen in liquid nitrogen, and stored at −80°C until use.

Using the above samples, we constructed four pooled RNA libraries. Total RNA was extracted from adipose tissues using the Trizol method according to the manufacturer's instructions (TaKaRa, Dalian, China). The RNA was treated with DNase I for 30 min at 37°C to remove residual DNA. Then, poly(A) mRNA was isolated using beads with oligo(dT). The purified mRNA was first fragmented to approximately 200 nt fragments using the RNA fragmentation kit. First-strand cDNA synthesis was performed using random hexamer primers and reverse transcriptase. The first strand cDNA was synthesized using the mRNA fragments as templates. After the first strand was synthesized, a custom second strand primer and strand synthesis buffer (Illumina) were both added, followed by dNTPs, RNase H and DNA polymerase I to nick translate the second-strand. The DNA product was then purified using a QiaQuick PCR column and eluted in EB buffer; the samples were then subjected to end repair, addition of a single A base, adaptor ligation, agarose gel isolation of approximately 200 nt cDNA, and PCR amplification. Then, the cDNA libraries were prepared according to Illumina's protocols and sequenced on the Illumina platform in BGI, Shenzhen, China.

### Read Mapping on the Bovine Reference Genome and Data Analysis

After the 3′ adaptor sequence was cleared away and redundant and low-quality reads were removed, the clean reads were aligned to the reference genome (http://hgdownload.soe.ucsc.edu/goldenPath/bosTau7/bigZips/bosTau7.fa.masked.gz) for assembly. Clean reads were 100% matched to the reference genome using the SOAPaligner program by not allowing any mismatches in reads. For the reads that could not be aligned with the reference sequences, we performed the SOAP alignment again while allowing less than 3 bp mismatches in the reads. A similar strategy was used to filter the unique sequence reads and multi-position reads. The gene expression level was normalized by calculating the number of reads per kilobase transcriptome per million mapped reads (RPKM) method. We calculated the fold change and P-value [decided by controlling the false discovery rate (FDR)] of each gene in two samples. Finally, the following criteria were used to judge the significance of gene expression difference: (1) Log2Ratio≥1 and (2) FDR≤0.001 [51]. All the basic data series were submitted to National Center for Biotechnology Information Gene Expression Omnibus with accession number GSE47653.

### Gene ontology and pathway enrichment analysis of DEGs

Gene ontology (GO) is an international standard gene functional classification system [52]. We first mapped the differentially expressed genes (DEGs) to GO terms in the database (http://www.geneontology.org/) to calculate gene numbers for every term and then used the corrected P-value≤0.05 as a threshold to find significantly enriched GO terms in the input list of DEGs. The P-value formula was as follows:
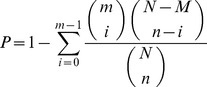
where N is the number of all genes with GO annotation; n is the number of DEGs in N; M is the number of all genes annotated to certain GO terms; and m is the number of DEGs in M. The calculated P-value was subjected to Bonferroni correction [50].

Next, KEGG (http://www.genome.jp/kegg/) was used to perform a pathway enrichment analysis of DEGs [53]. The calculation formula was the same as that in the GO analysis. Here, N is the number of all genes with KEGG annotation, n is the number of DEGs in N, M is the number of genes annotated to a specific pathway, and m is the number of DEGs in M. Biological pathways with an FDR≤0.05 were considered significant.

### Identification of novel transcribed units

To discover novel transcribed regions, we compared our assembled transcripts and annotated genomic transcripts from a reference annotated sequence. The assembled transcripts that did not align with the reference transcripts were then aligned with the reference genome. To be defined as a novel transcript, an assembled transcript must meet three requirements: (a) the transcript must be at least 200 bp away from an annotated gene, (b) the transcript has a length of over 180 bp, and (c) the sequencing depth is no less than 2. After discovering novel transcripts, we used the Coding Potential Calculator (CPC, http://cpc.cbi.pku.edu.cn/) to assess the protein-coding potential of the novel units.

### Analysis of alternative splicing

We used the SOAPsplice software to detect alternative splicing (AS) events. First, all of the reads were mapped to the reference genome for an intact alignment. Then, the unmapped reads were prepared for the next spliced alignment using the spliced alignment algorithm and the splice junctions were reported. Finally, we used both the mapping results and the known splice junctions to detect seven AS events according to Wang et al [42].

### Detection of Putative SNPs

We used SOAPsnp software to detect SNPs in the four samples based on the massively parallel Illumina GA technology. SOAPsnp is a member of the SOAP (Short Oligonucleotide Analysis Package) software [54]. The program calculates the likelihood of each genotype at each site based on the alignment of short reads to a reference sequence together with the corresponding sequencing quality scores. It then infers the genotype with highest posterior probability at each site based on Bayes' theorem (the reverse probability model). Thus, we have taken into account the intrinsic bias or errors that are common in Illumina GA sequencing data and recalibrated the quality values for use in inferring consensus sequence. The SNP detection stringency conditions were as follows: (1) at least two unique mapping reads that support the polymorphic nucleotide and (2) quality score ≥20.

### Validation of differentially expressed genes

To validate the sequence results, 15 genes that were specially or highly expressed in one of the four samples were selected and normalized to β-actin via the quantitative real-time PCR (qRT-PCR) method. Total RNA (1.0 µg from tested tissues) was reverse-transcribed to cDNA using the PrimeScript RT reagent Kit with gDNA Eraser according to the manufacturer's instructions. Primers were designed and are shown in Table_S13. qRT-PCR was performed using a Bio-Rad CFX 96 Real Time Detection System and SYBR Green PCR Master Mix in a 20 µl reaction. The relative gene expression values were calculated using the 2^−ΔΔCt^ method.

## Supporting Information

Figure S1 **The expression of genes in the four adipose tissue patterns detected by RT-qPCR and RNA-seq.**
(DOC)

Figure S2 **GO analysis for the other 46 genes expressed over 500 RPKM.**
(TIF)

Figure S3 **Genes changed in the PPAR signaling pathway from fetal to adult period.**
(TIF)

Table S1 **Genes commonly and highly expressed in the four adipose tissues.**
(XLS)

Table S2 **Gene Ontology assignment for diferentially expressed gene between adipose tissue of fetal bovine and adiose tissue of adult bovine.**
(XLS)

Table S3 **Gene Ontology assignment for diferentially expressed gene between adipose tissue of adult bovine and adiose tissue of adult steer.**
(XLS)

Table S4 **Gene Ontology assignment for diferentially expressed gene between adipose tissue of adult bovine and adiose tissue of adult heifer.**
(XLS)

Table S5 **Gene Ontology assignment for diferentially expressed gene between adipose tissue of adult bovine and adiose tissue of adult steer.**
(XLS)

Table S6 **List of KEGG pathway categories for differentially expressed genes between adipose tissue of fetal bovine and adiose tissue of adult bovine.**
(XLS)

Table S7 **List of KEGG pathway categories for differentially expressed genes between adipose tissue of adult bull and adiose tissue of adult steer.**
(XLS)

Table S8 **List of KEGG pathway categories for differentially expressed genes between adipose tissue of adult bull and adiose tissue of adult heifer.**
(XLS)

Table S9 **List of KEGG pathway categories for differentially expressed genes between adipose tissue of adult heifer and adiose tissue of adult steer.**
(XLS)

Table S10 **List of the putative SNPs in adipose tissue of fetal bovine.**
(XLS)

Table S11 **List of the putative SNPs in adipose tissue of adult bull.**
(XLS)

Table S12 **List of the putative SNPs in adipose tissue of adult heifer.**
(XLS)

Table S13 **List of the putative SNPs in adiose tissue of adult steer.**
(XLS)
