# Correction: Characterization of Transcriptional Complexity during Adipose Tissue Development in Bovines of Different Ages and Sexes

**DOI:** 10.1371/journal.pone.0113881

**Published:** 2014-11-12

**Authors:** The PLOS ONE Staff

Table 2 is incorrect. Please see the corrected Table 2 here.

**Table 2:** RNA-Seq gene expression results for the four patterns of adipose tissues.

Category	ATFB	ATAB	ATAH	ATAS
Highly expressed genes♯	184	181	169	167
Medium expressed genes§	5,075	5,443	6,039	6,119
Lowly expressed genes∮	6,238	5,850	5,423	5,335
Total expressed genes	11,497	11,474	11,631	11,621
Unexpressed genes	736	759	602	612

Note: The total number of genes are the sum of the data in the columns total expressed genes and unexpressed genes.

ATFB: adipose tissues of fetal bovines; ATAB: adipose tissues of adult bulls; ATAH: adipose tissues of adult heifers; ATAS: adipose tissues of adult steers.

♯ : Gene expressional level ≥ 500 RPKM.

§ : Gene expressional level ranging from 10 to 500 RPKM.

^∮^: Gene expressional level < 10 RPKM.
