# Supplementary material for: Characterization of Transcriptional Complexity during Adipose Tissue Development in Bovines of Different Ages and Sexes
Source: PLoS One. 2014 Jul 1;9(7):e101261. doi: 10.1371/journal.pone.0101261 (PMC4077742; doi:10.1371/journal.pone.0101261)
Supplement: Figure S1 — The expression of genes in the four adipose tissue patterns detected by RT-qPCR and RNA-seq. (DOC) [file pone.0101261.s001.doc]

**
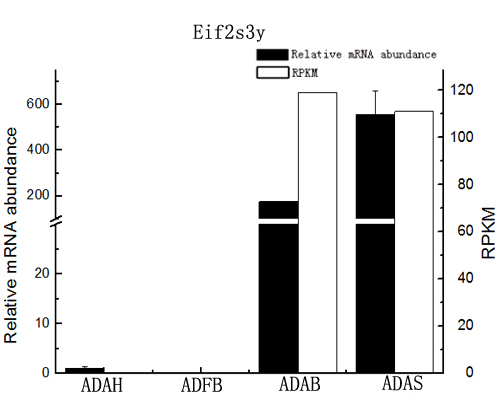

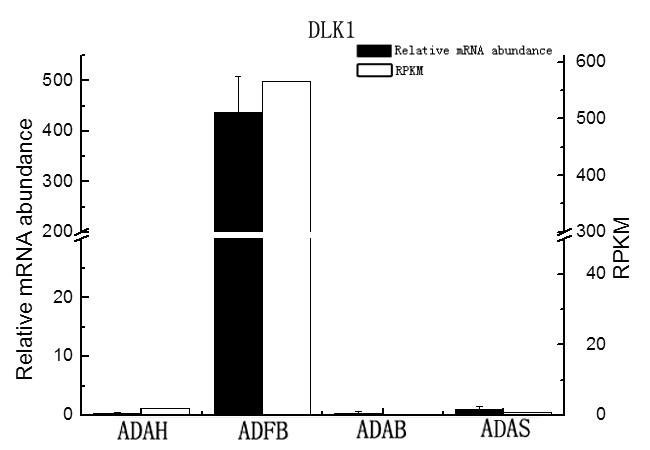

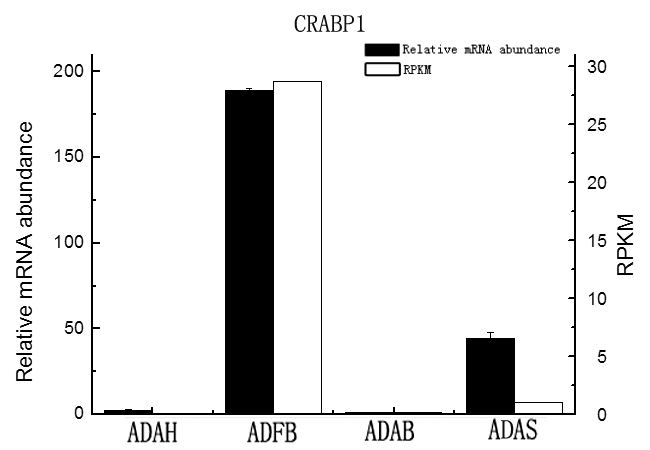

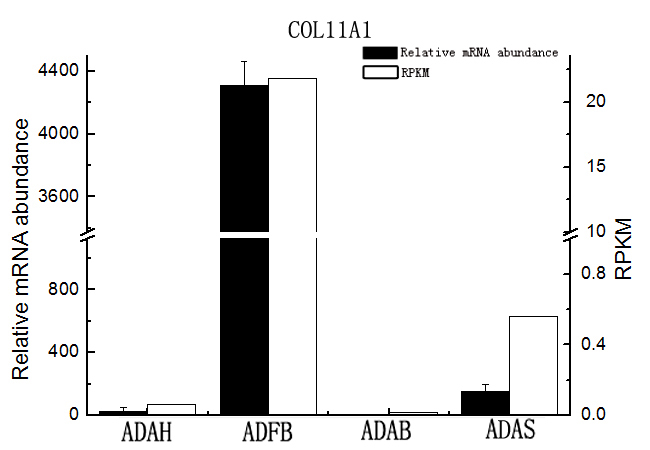

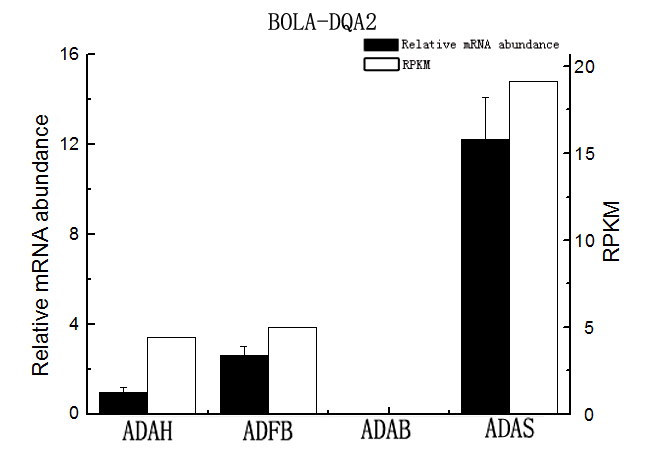

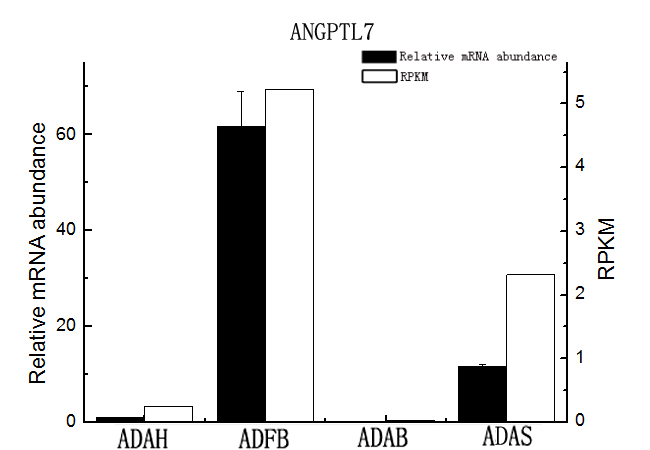

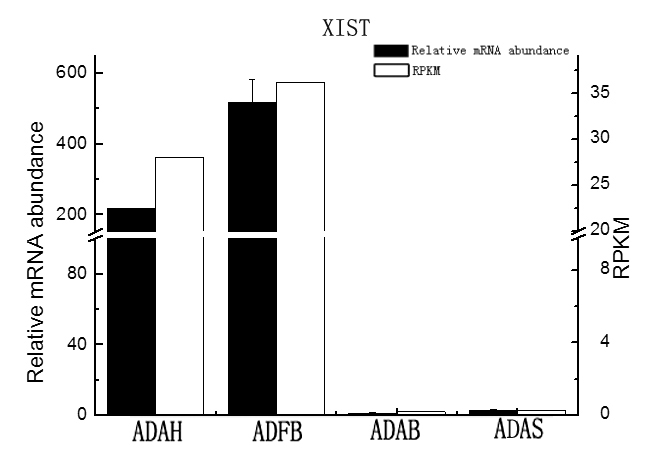

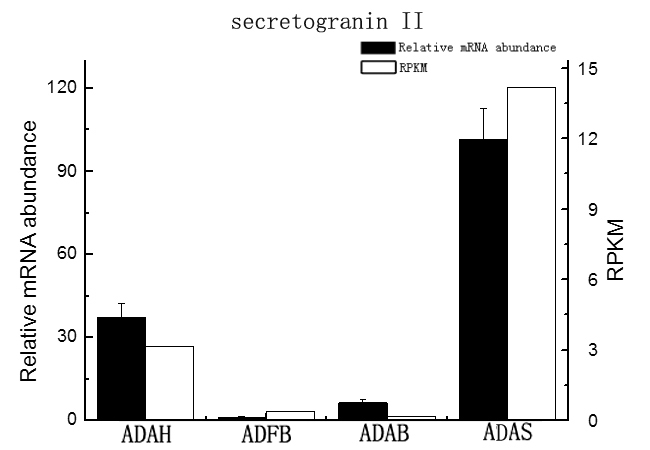

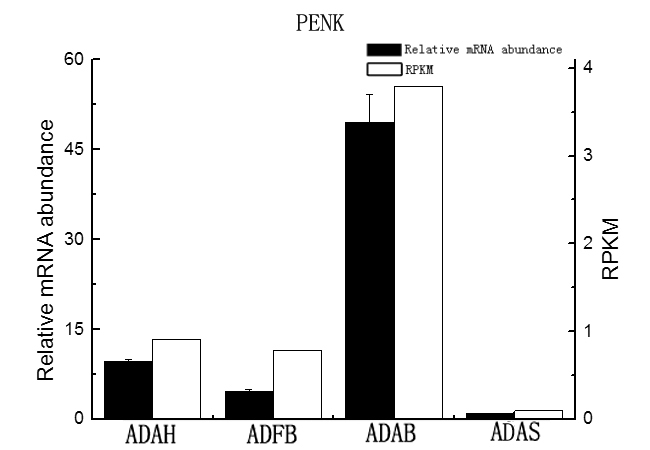

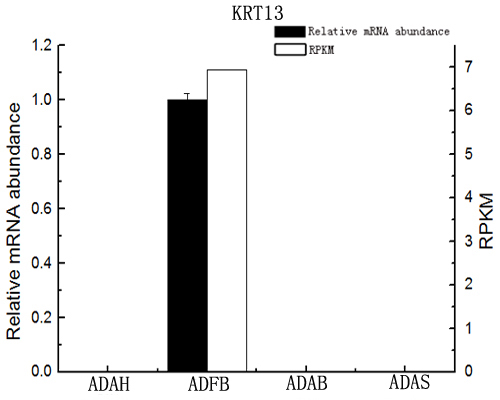

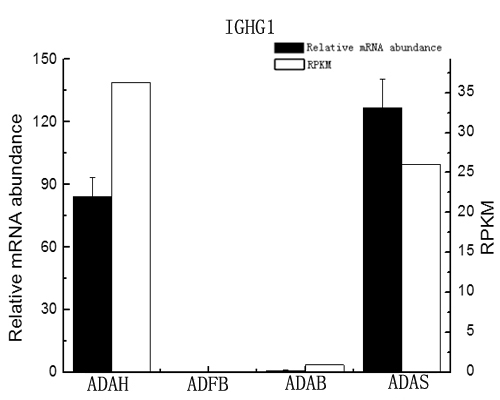

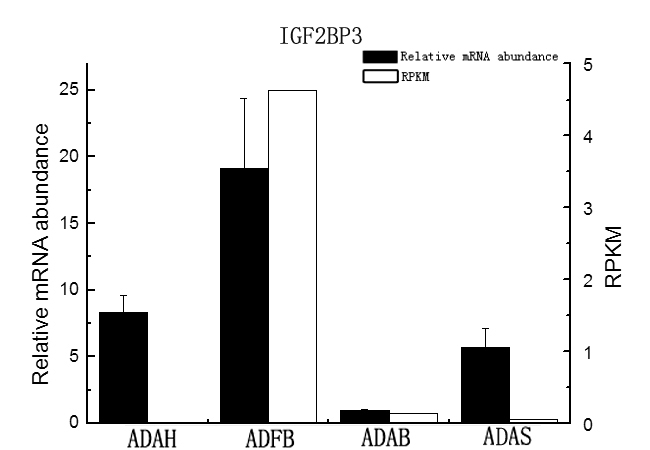

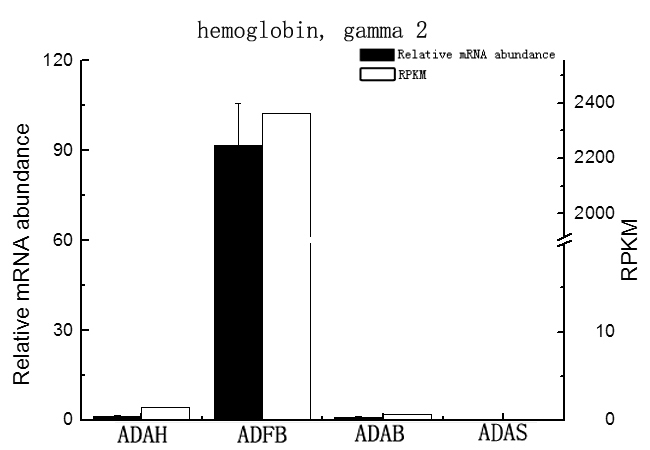

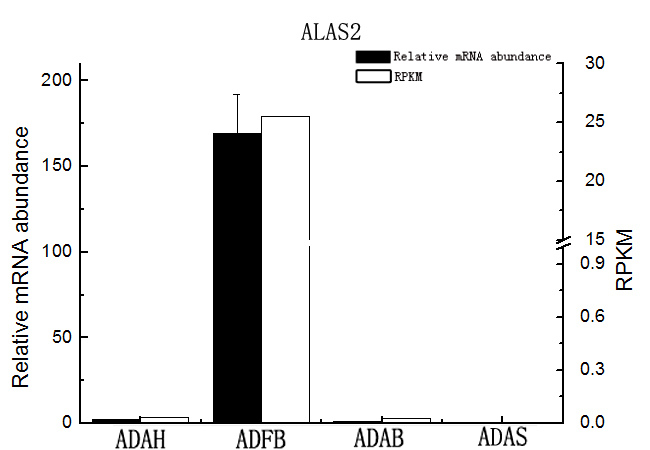

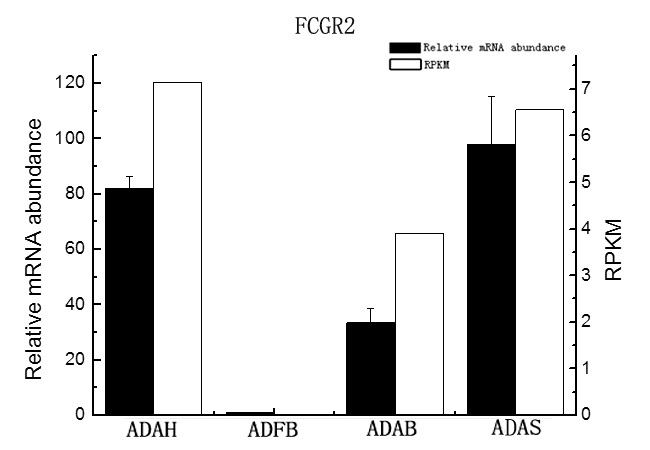
**

**Figure S1 The expression of genes in the four adipose tissue patterns detected by RT-qPCR and RNA-seq.**
