# Supplementary figures and images for: Characterization of Transcriptional Complexity during Adipose Tissue Development in Bovines of Different Ages and Sexes
Source: PLoS One. 2014 Jul 1;9(7):e101261. doi: 10.1371/journal.pone.0101261 (PMC4077742; doi:10.1371/journal.pone.0101261)

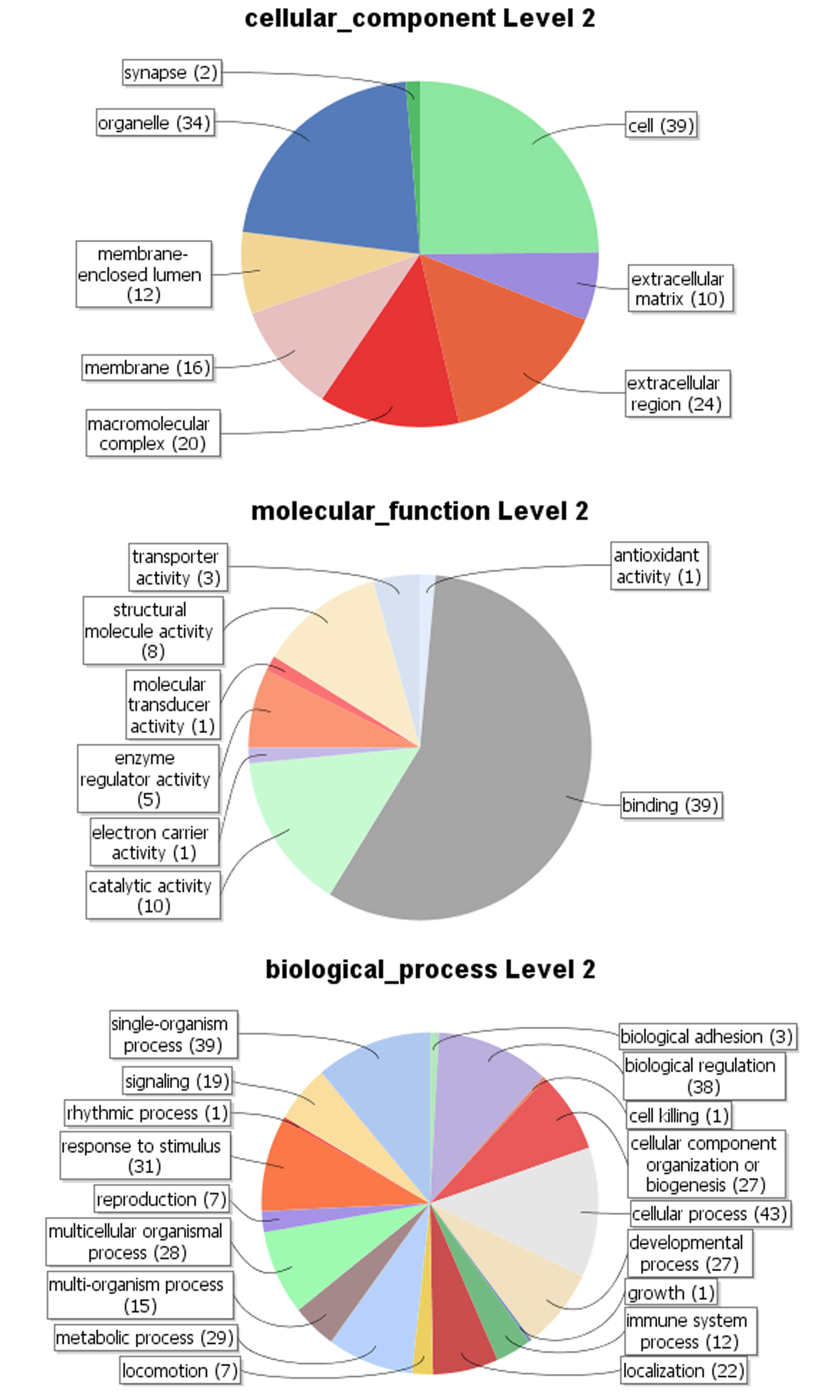

Supplement: Figure S2 — GO analysis for the other 46 genes expressed over 500 RPKM. (TIF) [file pone.0101261.s002.tif]

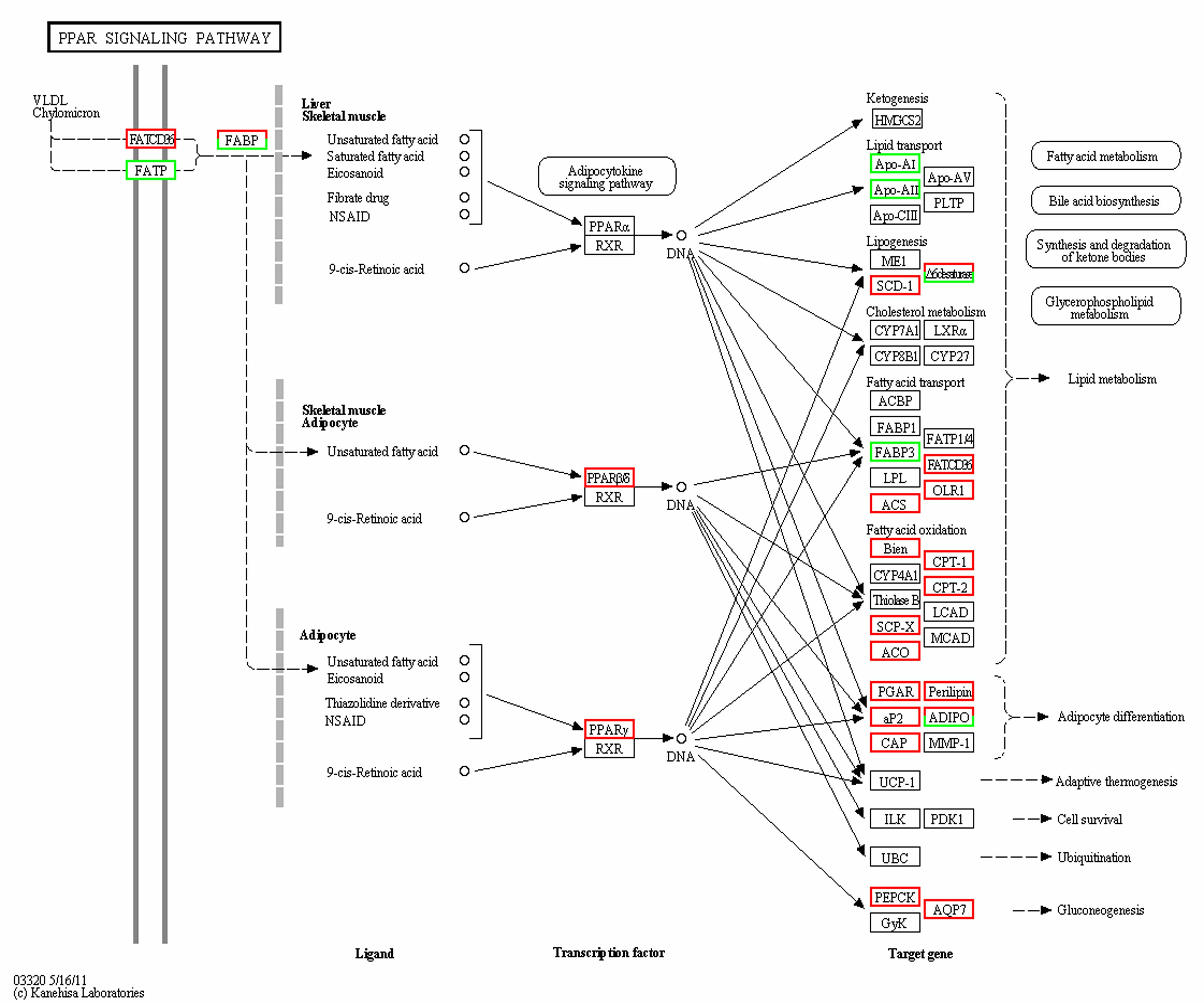

Supplement: Figure S3 — Genes changed in the PPAR signaling pathway from fetal to adult period. (TIF) [file pone.0101261.s003.tif]
